# Hypoglycemic Activity of Aqueous Root Bark Extract* Zanthoxylum chalybeum* in Alloxan-Induced Diabetic Rats

**DOI:** 10.1155/2016/8727590

**Published:** 2016-03-16

**Authors:** Moses Solomon Agwaya, Peter California Vuzi, Agnes Masawi Nandutu

**Affiliations:** ^1^Natural Chemotherapeutics Research Institute, Ministry of Health, Kampala, Uganda; ^2^Department of Biochemistry and Sports Science, College of Natural Sciences, Makerere University, Kampala, Uganda

## Abstract

*Background*. Medicinal plants offer cheaper and safer treatment options to current diabetic drugs. The present study evaluated the effect of aqueous root bark extract of* Zanthoxylum chalybeum *on oral glucose tolerance and pancreas histopathology in alloxanized rats.* Method*. Diabetes was induced in rats by administration of alloxan monohydrate. Root extract of* Z. chalybeum *was administered to rats at 200 and 400 mg/kg BW daily for 28 days. Blood glucose was measured by glucometer and pancreatic histopathology evaluated microscopically.* Results*. Initial increase was observed in blood glucose of the rats after oral administration of glucose from time zero. Two hours after treatment with* Z. chalybeum*, a significant reduction in blood glucose was observed within treatment groups (*p* < 0.05) compared to 0.5 hr and 1 hr. There was no significant difference between treatment group receiving 400mg/Kg BW extract and the normal groups (*p* = 0.27), implying that the former group recovered and were able to regulate their blood sugar, possibly via uptake of glucose into cells. The reversal in pancreatic histopathology further supports the protective effect of* Z. chalybeum *extract towards diabetic damage.* Conclusion*. Extract of* Z. chalybeum *is effective in controlling blood glucose in diabetes and protecting pancreatic tissues from diabetic damage.

## 1. Introduction

Diabetes mellitus is a group of metabolic diseases characterized by elevated blood glucose levels resulting from defects in insulin secretion, insulin action, or both [1]. The chronic hyperglycemia of diabetes is associated with long-term damage, dysfunction, and failure of various organs, especially the eyes, kidneys, nerves, heart, and blood vessels [2]. Diabetes is a major risk factor for the development of cardiovascular disease. More than 70% of deaths in diabetic patients are due to vascular disease. One of the greatest factors in the development and progression of the complications of diabetes mellitus is hyperglycemia [3]. Hyperglycemia contributes to majority of diabetic complications by altering vascular cellular metabolism, vascular matrix molecules, and circulating lipoproteins [2].

One method used for testing for diabetes is the oral glucose tolerance test. The oral glucose tolerance test (OGTT) measures the body's ability to use glucose which is the main source of energy for body cells.

Treatment of diabetes involves use of drugs that reduce glucose levels, including insulin and oral antihyperglycemic drugs. Although there is treatment for diabetes mellitus, most drugs in current use are seriously constrained by both their side effects and cost of treatment. Due to these challenges, populations mainly in Sub-Saharan Africa have resorted to cheaper and readily available alternative sources of treatment, such as use of medicinal plants or traditional medicines [4]. The World Health Organization (WHO) estimates that 80% of the worlds' populations use traditional medicine. The continued use of traditional medicines is linked to their low cost and a general belief that they have minimal side effects [5].

*Zanthoxylum chalybeum* which belongs to the family Rutaceae is a traditional medicinal plant commonly used by some tribes in Eastern Uganda for treatment of malaria and diabetes [6]. It is a deciduous spiny shrub that grows up to a height of 12 m, with a rounded but open crown. The bole has characteristically large, conical, woody knobs with sharp thorns. The branches also bear scattered thorns with conspicuous dark scales. It has compound leaves comprising 3 to 5 pairs of shiny leaflets plus a terminal leaflet, with a strong citrus smell when crushed [6]. Stem bark decoctions or root bark decoctions are widely taken to treat diabetes, malaria, sickle cell disease, ulcers, and tumors. Root bark decoctions are considered stronger than stem bark decoctions [6, 7].

However, although* Z. chalybeum* is documented to be used in management of diabetes mellitus in traditional medicine, no scientific study has been done to validate its hypoglycemic activity. This study is the first to evaluate the antidiabetic activity of* Z. chalybeum* using a laboratory rat model in order to provide scientific evidence to support its use by local communities in the management and treatment of diabetes.

## 2. Material and Methods

### 2.1. Material

*Zanthoxylum chalybeum* root bark was obtained from Katakwi district in Eastern Uganda where it is abundant and locally called* Eusuk* (Ateso local language). The plant was authenticated by a plant taxonomist at Natural chemotherapeutics Research Institute, Ministry of Health. After collection a voucher specimen was deposited at the National Herbarium, Makerere University, Kampala. The root was washed, debarked, and dried in an air oven at 50°C for 48 h. The bark was then pulverized into powder using a grinder. The powder was extracted by boiling in water for 30 minutes and allowing cooling to room temperature. The extract was then filtered and concentrated using an air oven at 50°C to obtain the crude extract. The crude extract was reconstituted in distilled water and used to evaluate the hypoglycemic property in rats.

### 2.2. Experimental Animals

Twenty-four male and 24 female Wistar albino rats (aged 12–14 weeks, weighing 180–240 g) were obtained from the College of Veterinary Medicine, Animal Resources and Biosecurity (COVAB), Makerere University, Kampala, and used for evaluation of the antidiabetic property of* Z. chalybeum* root bark extract in rats.

### 2.3. Induction of Diabetes in Rats

The animals were allowed to acclimatize for 2 weeks, and then diabetes mellitus was chemically induced in the rats using freshly prepared solution of alloxan monohydrate in distilled water at a dose of 150 mg Kg^−1^ BW injected intraperitoneally. Since alloxan caused fatal hypoglycemia due to massive insulin release by the pancreas, the rats were in addition orally given 20% (w/v) glucose solution (10 mL) after 6 h. They were further kept for 24 h on 5% (w/v) glucose solution to prevent hypoglycemia. Rats which developed diabetes mellitus observed by glycosuria and hyperglycemia (i.e., blood glucose concentration >250 mgdL^−1^) were selected for the subsequent experimental tests. This study was approved by Graduate Research Committee, College of Natural Sciences, Makerere University, Kampala.

### 2.4. Experimental Design

Animals were kept under standard laboratory conditions (25 ± 3°C, 12 h light/dark cycle) and had free access to food and clean tap water* ad libitum* for 28 days of experimental period. The procedures were of animal experiments in accordance with the Organization for Economic Cooperation and Development (OECD) guidelines for testing of chemicals [8]. All the procedures were in accordance with the International Council for Laboratory Animal Science (ICLAS) and Ethical Guideline for Researchers. The experimental study was conducted on four groups of animals each with twelve rats. Six male and six female Wistar albino rats were randomly allocated to each of the four groups. The groups were treated as follows: Group I consisted of diabetic rats orally given water, food, and metformin by gavage (10 mg/Kg BW) once daily for 28 days. Group II consisted of diabetic rats orally given water, food, and* Z. chalybeum* extract by gavage (200 mg/Kg BW) once daily for 28 days. Group III consisted of diabetic rats orally given water, food, and* Z. chalybeum* extract by gavage (400 mg/Kg BW) once daily for 28 days. Group IV consisted of normal rats, orally given water and food with no treatment administered, that is, the normal control group.

### 2.5. Effect of* Zanthoxylum chalybeum* on Oral Glucose Tolerance in Alloxan-Induced Diabetic Rats

After 28 days of repeated treatment but before sacrifice, two male and two female Wistar albino rats were randomly selected from each of the four groups and blood samples were collected from tail vein of the rats of control and treated groups after an overnight fast to obtain baseline blood glucose levels. Subsequently, rats of both control and treated groups were orally given glucose (2 g/Kg BW) by gavage. Blood samples were collected from tail vein of the rats at intervals of 30 min up to 2 h for estimation of glucose concentrations using a glucometer.

### 2.6. Histopathological Analysis of Rat Tissues following Treatment

The animals were anesthetized and the pancreas was removed and fixed in a 10% solution of formaldehyde. The tissues were dehydrated because the reagents used at a later stage were immiscible with water. Varying concentration of isopropyl alcohol, that is, 70%, 80%, 90%, 96%, and 100%, was used for the dehydration. The minimum time for dehydration between two different concentrations was 1 h. The fixed tissues were then cleared in xylene and embedded in paraffin wax. The sections (5 *μ*m) from each of the tissues were examined using a light microscope (×40) after staining with hematoxylin and eosin dye.

### 2.7. Statistical Data Analysis

The data was analyzed using Graphpad software. The glucose concentration results were expressed as mean ± Standard Error of the Mean (SEM). The mean and SEM of the treatment groups were generated by use of the analysis of variance (ANOVA) test. The significant difference between and within the treatment groups was considered significant at set *p* value < 0.05 using Dunnett's multiple comparison tests.

## 3. Results

### 3.1. Effect of* Z. chalybeum* Root Bark Extract on Oral Glucose Tolerance in Diabetic Rats

**Figure 1:**
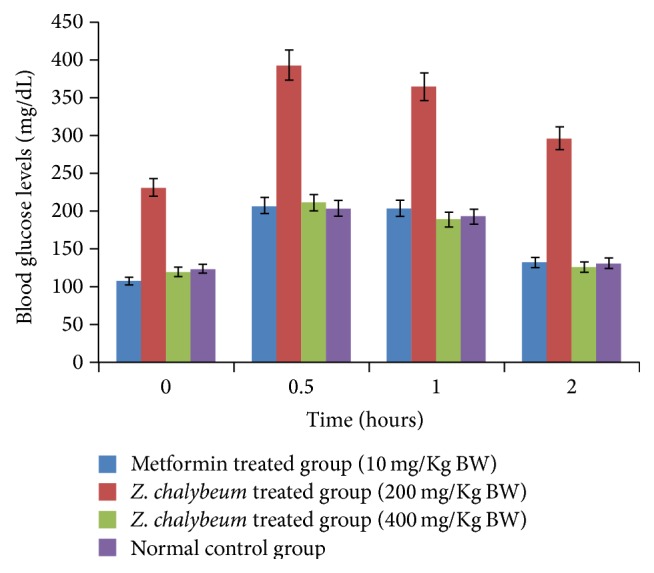
Effect of* Z. chalybeum* root bark extract on oral glucose tolerance in diabetic rats.

Oral glucose tolerance test was carried out 28 days after repeated doses of the aqueous root extract. There was a significant increase in blood sugar level after oral dosing within the same treatment group compared to treatment at 0 hr (Figure 1). Two hours following treatment with the extract, there was a significant reduction in blood glucose within the same treatment group compared to that after 0.5 h and 1 hr. However, there was no significant difference between the treatment group receiving 400 mg/Kg BW and the control groups after two-hour treatment. The results further show that diabetic rats receiving* Z. chalybeum aqueous root* extract (400 mg/Kg BW) made some recovery and were able to regulate their sugar levels (Figure 1).

**Figure 2:**
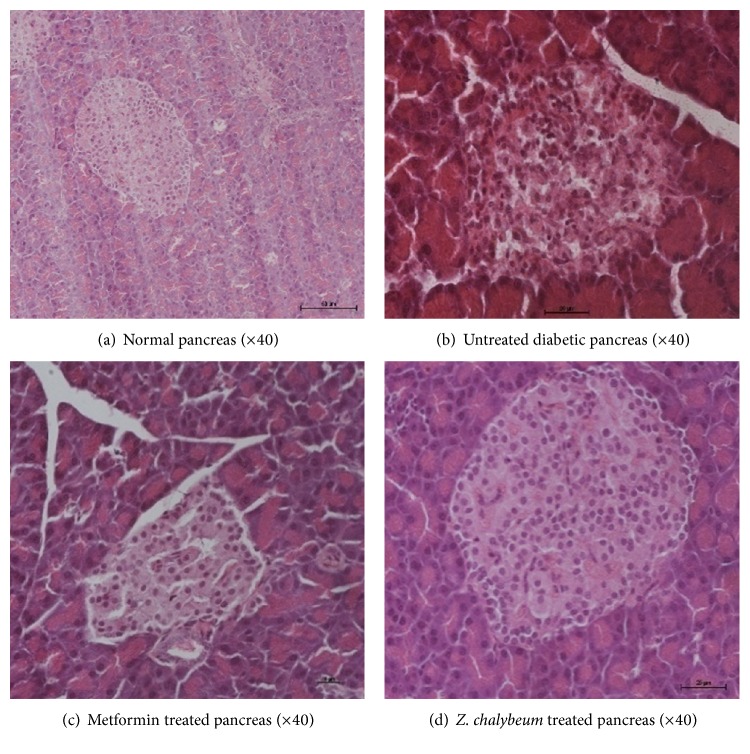
Effect of* Z. chalybeum* aqueous root bark extract on the histology of the pancreas in diabetic rats.

Induction of diabetes with alloxan resulted in severe damage to the *β*-cells of the islets of Langerhans (Figure 2(a)). A month after treatment with* Z. chalybeum* (400 mg/Kg BW), there was regeneration of the central *β*-cells (Figure 2(d)). The normal structure of the cells and the structure of the islets were restored. There was also increase in the number of secretive *β*-cells. Beta cells are epithelial cells and have the ability to regenerate. The* Z. chalybeum* extract stimulates the regeneration of *β*-cells of the islets. However the number of *β*-cells was still low and yet the animals were able to maintain glucose levels near normal. This therefore suggests that the* Z. chalybeum* extract also increases the sensitivity of the insulin receptors to the effects of insulin.

## 4. Discussion

Diabetes mellitus causes disturbances in the uptake of glucose by cells as well as glucose metabolism. Thus, alloxan-induced hyperglycemia is a very useful experimental way of studying and demonstrating the activity of new hypoglycemic agents [9]. Oral glucose tolerance tests were used to analyze blood glucose levels taken at different regular intervals after repeated treatments with* Z. chalybeum* aqueous root extracts. Results of the oral glucose tolerance test, using aqueous root extract of* Z. chalybeum* (400 mg/Kg BW), indicate significant decrease (*p* < 0.05) in blood glucose levels of the alloxan-induced diabetic rats (Figure 1). This suggests that the aqueous root extracts of* Z. chalybeum* enhance glucose utilization and hence improve glucose tolerance in diabetic rats. This also reaffirms that glucose tolerance test (GTT) is a suitable measure and indicator for the cells ability to use glucose, the body's main source of energy [10]. OGT test can thus be used to diagnose prediabetic and diabetic conditions. Similarly, oral administration of aqueous root extract of* Z. chalybeum* (400 mg/Kg BW) had a significant glucose lowering effects in alloxan-induced diabetic rats (*p* < 0.05).

Other species in the genus* Zanthoxylum* have been studied experimentally and reported to have significant antidiabetic activity. For example, various parts of* Z. zanthoxyloides* including the roots, bark, and leaves have been used for medicinal purposes, including the treatment of diabetes mellitus.* Z. zanthoxyloides* has been reported to significantly (*p* < 0.05) lower blood glucose in treated animals in comparison to nontreated groups [11]. Other species in the genus that are used traditionally to treat diabetes in India are* Z. armatum* and* Z. nitidum* [12, 13]. The beneficial effects of* Z. chalybeum* treatment in diabetic rats in this study were likely due to improved insulin release and glucose uptake in remnant *β*-cells.

The induction of diabetes using alloxan resulted in severe damage of *β*-cells of the islets of Langerhans (Figure 2(b)). However, after repeated treatment with* Z. chalybeum* (400 mg/Kg BW), for 28 days, there was regeneration of the central *β*-cells (Figure 2(d)). There was also a notable increase in the number of secretive *β*-cells which are epithelial cells with ability to regenerate. The* Z. chalybeum* extract appeared to stimulate the regeneration of *β*-cells of the islets of Langerhans. Albeit the still low number of the *β*-cells, the animals were able to maintain glucose levels close to the normal. This therefore also implies that the* Z. chalybeum* extract increases the sensitivity of the insulin receptors to insulin.

Previous reports indicate that medicinal plants that possess hypoglycemic activity act through various mechanisms including improvement in the sensitivity of target cells to the effects of insulin, augmenting glucose-dependent insulin secretion, and stimulating the regeneration of *β*-cells of islets of Langerhans in pancreas of alloxan-induced diabetic rats [14]. Some of the medicinal plants seem to regulate enzymes of glycolysis, gluconeogenesis, and other pathways [15]. Active phytochemical compounds act through a variety of mechanisms; however, in the present study, identification of the mechanism of action of the extract was not done; thus the suggestions made are only hypothetical.

Earlier studies on phytochemical analysis of aqueous roots extract of* Z. chalybeum* reported presence of the following compounds: tannins, reducing sugars, saponins, alkaloids, coumarin derivatives, flavonoides, steroid glycosides, triterpenes, and anthocyanosides [16]. Presence of flavonoids, steroids, terpenoids, and phenolic acids has been suggested by several authors to be responsible for antidiabetic activity [17]. Flavonoids have also been known to regenerate the damaged beta cells in alloxan-induced diabetic rats and act as insulin secretagogues [18]. Thus, the hypoglycemic activity of aqueous roots extract of* Z. chalybeum* may be due to the presence of hypoglycemic flavonoids, terpenes, or saponins; however, this also requires further investigation. The antihyperglycemic effect of* Z. chalybeum* may be attributed to the potentiation of insulin from existing *β*-cells of the islets of Langerhans. The blood glucose lowering effect of* Z. chalybeum* was compared with that of metformin, a standard drug which has been in use for many years for treatment of diabetes and acts by stimulating insulin secretion from pancreatic *β*-cells [18].

## 5. Conclusion

Study has shown that oral administration of aqueous root bark extract of* Z. chalybeum* to alloxan-induced diabetic rats improves their glucose tolerance, an important finding in the control of diabetes. This suggests that* Z. chalybeum* is useful in protection and amelioration of diabetic complications through enhancement of regeneration of *β*-cells of the islets of Langerhans. Therefore, the effects of* Z. chalybeum* appear to be curative rather than palliative. Further studies are needed to define the active agents present and their mode of activity.

## Abbreviations

OGTT: Oral glucose tolerance test
WHO: World Health Organization
COVAB: College of Veterinary Medicine, Animal Resources and Biosecurity
OECD: Organization for Economic Cooperation and Development
ICLAS: International Council for Laboratory Animal Science
SEM: Standard Error of the Mean
ANOVA: Analysis of variance
Kg BW: Kilogram body weight.

## References

[1] Khan A., Zaman G., Anderson R. A. (2009). Bay leaves improve glucose and lipid profile of people with Type 2 diabetes. *Journal of Clinical Biochemistry and Nutrition*.

[2] American Diabetes Association (2010). Diagnosis and classification of diabetes mellitus. *Diabetes Care*.

[3] Fiorentino T. V., Prioletta A., Zuo P., Folli F. (2013). Hyperglycemia-induced oxidative stress and its role in diabetes mellitus related cardiovascular diseases. *Current Pharmaceutical Design*.

[4] Modak M., Dixit P., Londhe J., Ghaskadbi S., Devasagayam T. P. A. (2007). Indian herbs and herbal drugs used for the treatment of diabetes. *Journal of Clinical Biochemistry and Nutrition*.

[5] Somani R., Kasture S., Singhai A. K. (2006). Antidiabetic potential of *Butea monosperma* in rats. *Fitoterapia*.

[6] Tabuti J. R. S., Schmelzer G. H., Gurib-Fakim A. (2011). *Zanthoxylum chalybeum* Engl. [Internet] record from PROTA4U. *Plant Resources of Tropical Africa*.

[7] Ogwang P. E., Nyafuono J., Agwaya M. S., Omujal F., Tumusiime R. H., Kyakulaga A. H. (2011). Preclinical efficacy and safety of herbal formulation for management of wounds. *Journal of African Health Sciences*.

[8] OECD. Organization for Economic Co-Operation and Development (2014). *OECD Guidelines for the Testing of Chemicals/Section 4: Health Effects Test No. 423; Acute Oral Toxicity-Acute Toxic Class Method*.

[9] Srinivasan K., Ramarao P. (2007). Animal models in type 2 diabetes research: an overview. *Indian Journal of Medical Research*.

[10] Ali M. A., Sultana M. C., Rahman B. M., Khatune N. A., Wahed M. I. I. (2015). Antidiabetic activity of ethanolic extract of *Semecarpus anacardium* (linn.) Stem barks in normal and alloxan induced diabetic rats. *International Journal of Pharmaceutical Science and Research*.

[11] Aloke C., Nwachukwu N., Ugwuja E. I. (2012). Effects of *Zanthoxylum zanthoxyloides* leaves on blood glucose, lipid profile and some liver enzymes in alloxan induced diabetic rats. *International Journal of Science and Nature*.

[12] Singh T. P., Singh O. M. (2011). Phytochemical and pharmacological profile of *Zanthoxylum armatum* DC.—an overview. *Indian Journal of Natural Products and Resources*.

[13] Arun K., Paridhavi M. (2012). An ethno botanical, phytochemical and pharmacological utilization of widely distributed species Zanthoxylum: a comprehensive over view. *International Journal of Pharmaceutical Invention*.

[14] Ayodhya S., Kusum S., Anjali S. (2010). Hypoglycemic activity of different extracts of various herbal plants. *International Journal of Research in Ayurveda & Pharmacy*.

[15] Arya A., Cheah S. C., Looi C. Y., Taha H., Mustafa M. R., Mohd M. A. (2012). The methanolic fraction of *Centratherum anthelminticum* seed down regulates pro-inflammatory cytokines, oxidative stress and hyperglycemia in STZ-nicotinamide-induced type 2 diabetic rats. *Food and Chemical Toxicology*.

[16] Nalule A. S., Mbaria J. M., Kimenju J. W. (2013). In vitro anti-helminthic potential and phytochemical composition of ethanolic and aqueous crude extracts of *Zanthoxylum chalybeum*. *African Journal of Pharmacy and Pharmacology*.

[17] Alagammal M., Agnel R. A., Mohan V. R. (2012). Anti-diabetic and anti-hyperlipidaemic effect of *Polygala javana* DC on alloxan induced diabetic rats. *International Research Journal of Pharmacy*.

[18] Mao-Ying Q.-L., Kavelaars A., Krukowski K. (2014). The anti-diabetic drug metformin protects against chemotherapy-induced peripheral neuropathy in a mouse model. *PLoS ONE*.

